# Phenotypic Plasticity of Mouse Spermatogonial Stem Cells

**DOI:** 10.1371/journal.pone.0007909

**Published:** 2009-11-19

**Authors:** Hiroko Morimoto, Mito Kanatsu-Shinohara, Seiji Takashima, Shinichiro Chuma, Norio Nakatsuji, Masanori Takehashi, Takashi Shinohara

**Affiliations:** 1 Department of Molecular Genetics, Graduate School of Medicine, Kyoto University, Kyoto, Japan; 2 Department of Development and Differentiation, Institute for Frontier Medical Sciences, Kyoto University, Kyoto, Japan; 3 Japan Science and Technology Agency, CREST, Kyoto, Japan; CNRS, France

## Abstract

**Background:**

Spermatogonial stem cells (SSCs) continuously undergo self-renewal division to support spermatogenesis. SSCs are thought to have a fixed phenotype, and development of a germ cell transplantation technique facilitated their characterization and prospective isolation in a deterministic manner; however, our in vitro SSC culture experiments indicated heterogeneity of cultured cells and suggested that they might not follow deterministic fate commitment in vitro.

**Methodology and Principal Findings:**

In this study, we report phenotypic plasticity of SSCs. Although c-kit tyrosine kinase receptor (Kit) is not expressed in SSCs in vivo, it was upregulated when SSCs were cultured on laminin in vitro. Both Kit^−^ and Kit^+^ cells in culture showed comparable levels of SSC activity after germ cell transplantation. Unlike differentiating spermatogonia that depend on Kit for survival and proliferation, Kit expressed on SSCs did not play any role in SSC self-renewal. Moreover, Kit expression on SSCs changed dynamically once proliferation began after germ cell transplantation in vivo.

**Conclusions/Significance:**

These results indicate that SSCs can change their phenotype according to their microenvironment and stochastically express Kit. Our results also suggest that activated and non-activated SSCs show distinct phenotypes.

## Introduction

Spermatogonial stem cells (SSCs) provide the foundation for spermatogenesis throughout the life of male animals [1], [2]. These cells produce differentiating cells and also maintain an undifferentiated state by undergoing self-renewal division. Despite their unique biology, the regulatory mechanism of SSC self-renewal has remained unclear. During the last decade, however, attempts have been made to characterize the surface phenotype of SSCs. Studies have established that SSCs express α6- and β1-integrin, GFRα1, CD9, Thy-1, and EpCAM but are negative for c-kit (Kit) or SSEA-1 [3]. Expression of these markers was analyzed using a germ cell transplantation technique transplanting cells freshly prepared from testes, because SSC activity, by definition, is examined only retrospectively after examining the characteristic of daughter cells [4]. These surface markers proved to be useful to purify SSCs in a deterministic manner by combining multiple parameters using cell sorter [5].

Recent studies revealed important functions of surface molecules on SSCs. For example, β1-integrins on SSCs play pivotal roles in migration into a germline niche after transplantation [6]. Another study also showed that GFRα1, which comprises a receptor for glial cell line-derived neurotrophic factor (GDNF), regulates SSC self-renewal. GDNF from Sertoli cells maintains SSCs in an undifferentiated state by binding to the GFRα1-c-ret receptor complex [7]. GFRα1 is expressed in a small population of undifferentiated spermatogonia, and changes in GDNF or GFRα1 levels can influence the fate of SSCs. For example, when GDNF is overexpressed in testes, spermatogenesis is impaired and clumps of undifferentiated spermatogonia accumulate in seminiferous tubules [7]. By contrast, a decrease in GDNF or GFRα1 level induces SSC differentiation and male infertility [7], [8]. In addition to GDNF, Sertoli cells secrete another cytokine, Steel factor (Sl). Sl binds to Kit on germ cells, and a lack of Sl-Kit interaction also results in impaired spermatogenesis [9]. However, Kit is not expressed in SSCs, but it promotes proliferation and suppresses apoptosis of differentiating spermatogonia [5], [9]–[11]. Nevertheless, the number of SSCs in Steel/Steel dickie (Sl^d^) mutant mice, which lack membrane-bound Sl, is reduced to ∼5% of wild-type (WT) mice. SSCs in these mice do not regenerate to the basal number, suggesting that Sl-Kit interaction influences SSC number in Sl^d^ mice [12]. Thus, how environmental stimuli influence SSCs in the decision between self-renewal and differentiation via surface molecules remains unclear.

In 2003, a long-term culture system for SSCs was reported [13]. Cultured SSCs, designated as germline stem (GS) cells, continued to proliferate for more than 2 years while maintaining stable genetic and epigenetic properties [14]. Development of this culture systems provided possibilities to study SSCs in vitro. However, the percentage of SSCs in GS cell culture was unexpectedly low, and only 0.04–1.26% could colonize and reconstitute seminiferous tubules of infertile animals [15]. Moreover, a variable proportion of the cells express Kit, suggesting that a majority of GS cells are differentiating spermatogonia. In contrast, transfection experiments suggested that a significant proportion of GS cells can act as SSCs. When GS cell clones were established by electroporation with a neo-resistant gene, ∼20% of picked GS cell colonies colonized seminiferous tubules and produced transgenic offspring [16]. These conflicting experiments suggest that SSC frequency is much higher than previous estimates by direct transplantation and also suggested that SSCs in vitro may exhibit properties that are distinct from those sustaining spermatogenesis in vivo.

In the present study, to clarify the phenotype of SSCs in vitro, we fractionated GS cells according to Kit expression, and examined the SSC activity using a germ cell transplantation technique. We found that GS cells show a constant level of SSC activity regardless of Kit expression. Kit was also strongly expressed in SSCs in vivo when they actively increase their number to colonize seminiferous tubules.

## Results

### Heterogeneity of GS Cells

**Figure 1:**
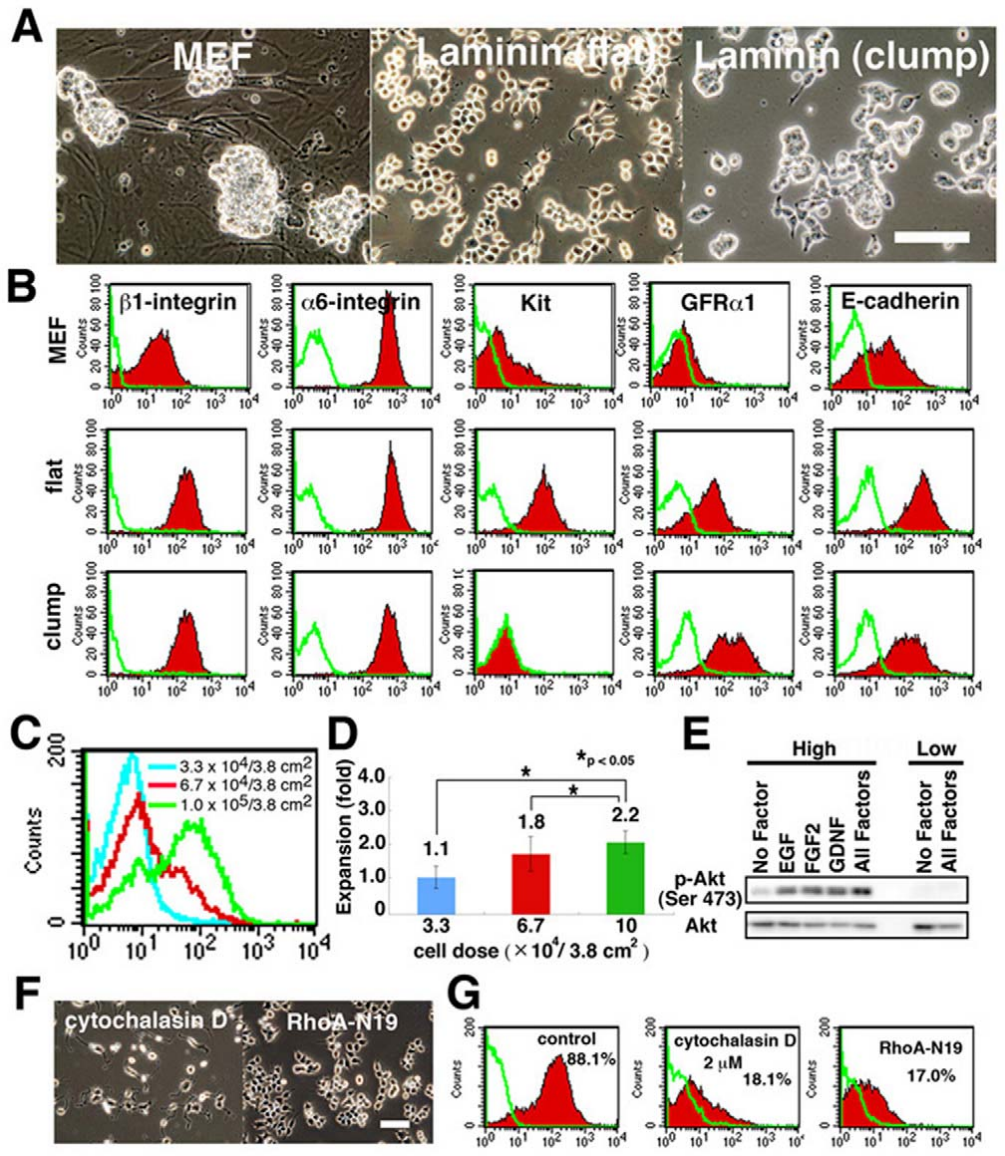
GS cells express Kit. (A) Morphological appearance. (B) FACS analysis of surface markers. Green line indicates the control. (C, D) Effect of cell density on Kit expression (C) and GS cell expansion (D). Cells were plated at the indicated density on laminin (n = 6). Values indicate the degree of expansion from the initially plated cells. (E) Western blot analysis of GS cells plated at 5×10^5^ or 3×10^4^ cells/9.5 cm^2^. (F, G) Appearance (F) and Kit expression (G) of GS cells after cytochalasin D treatment or transfection of RhoA-N19 cDNA. Bar = 100 µm (A, F).

We previously reported that a significant proportion of GS cells express Kit [13]. We therefore assumed that SSCs would be enriched by removing Kit^+^ cells from the culture, because Kit is expressed in differentiating spermatogonia. However, Kit expression in mouse embryonic fibroblast (MEF)-based GS cell culture varied depending on the timing of analysis, and we could not get consistent results. On the other hand, GS cells proliferate for long periods on laminin-coated dishes [15]. GS cells on laminin differ from those on MEFs in colony morphology. Although they form three-dimensional clump-like colonies similar to GS cells on MEFs, they can also form two-dimensional flat colonies (Figure 1A). When these cells were analyzed by flow cytometry, they were different from those on MEF in Kit expression levels (Figure 1B). Whereas the percentage of Kit-expressing cells increased up to ∼90% in the flat colony, clump-type colonies showed little or no Kit expression. In both conditions, >95% of the cultured cells remained viable.

Of the several factors examined (laminin concentration, incubation time, and temperature), we found that the development of two kinds of colonies was most strongly influenced by plating density (Figure 1C). When cells were plated at 1×10^5^ cells/3.8 cm^2^, 60–90% of the cultured cells showed Kit expression. However, very little expression was observed when cells were plated at <3.3×10^4^/3.8 cm^2^. Seeding density also had an impact on GS cell proliferation, and GS cells in clump-like colonies did not proliferate as much as did those in fibroblast-like colonies (Figure 1D). Consistent with this observation, Akt, which promotes GS cell proliferation [3], [17], [18], was strongly phosphorylated when GS cells were plated at the higher cell density (Figure 1E).

Using two different cell densities (1 ×10^5^ and 3.3×10^4^ cells/cm^2^), we analyzed the expression of other cell surface markers by flow cytometry (Figure 1B). Although the two types of cells showed a significant difference in Kit expression level, GFRα1, a marker for A single (A_s_) and A paired (A_pr_) spermatogonia, and E-cadherin, a marker for undifferentiated spermatogonia [3], were expressed at comparable levels regardless of the type of colonies. We did not find significant changes in other spermatogonia or SSC markers, including α6- and β1-integrins.

Because a difference in cell shape implicated changes in cytoskeletal tension [19], we checked whether actin cytoskeleton was involved in Kit expression by adding actin-disrupting cytochalasin D. Cytochalasin D not only changed the shape of GS cells but it also decreased Kit expression (Figure 1F and G). Because small G proteins are central regulators of cell contractility, we also checked the effect of small G proteins by producing GS cells that stably express Rac, RhoA, and cdc42 dominant-negative mutants. Although no apparent morphological differences were noted among transfectants, dominant-negative RhoA mutants clearly decreased Kit expression (Figure 1F and G). These results suggested that cytoskeletal tension plays an important role in regulation of Kit expression.

### Analysis of Kit Function in GS Cell Self-Renewal and Homing into Niche

**Figure 2:**
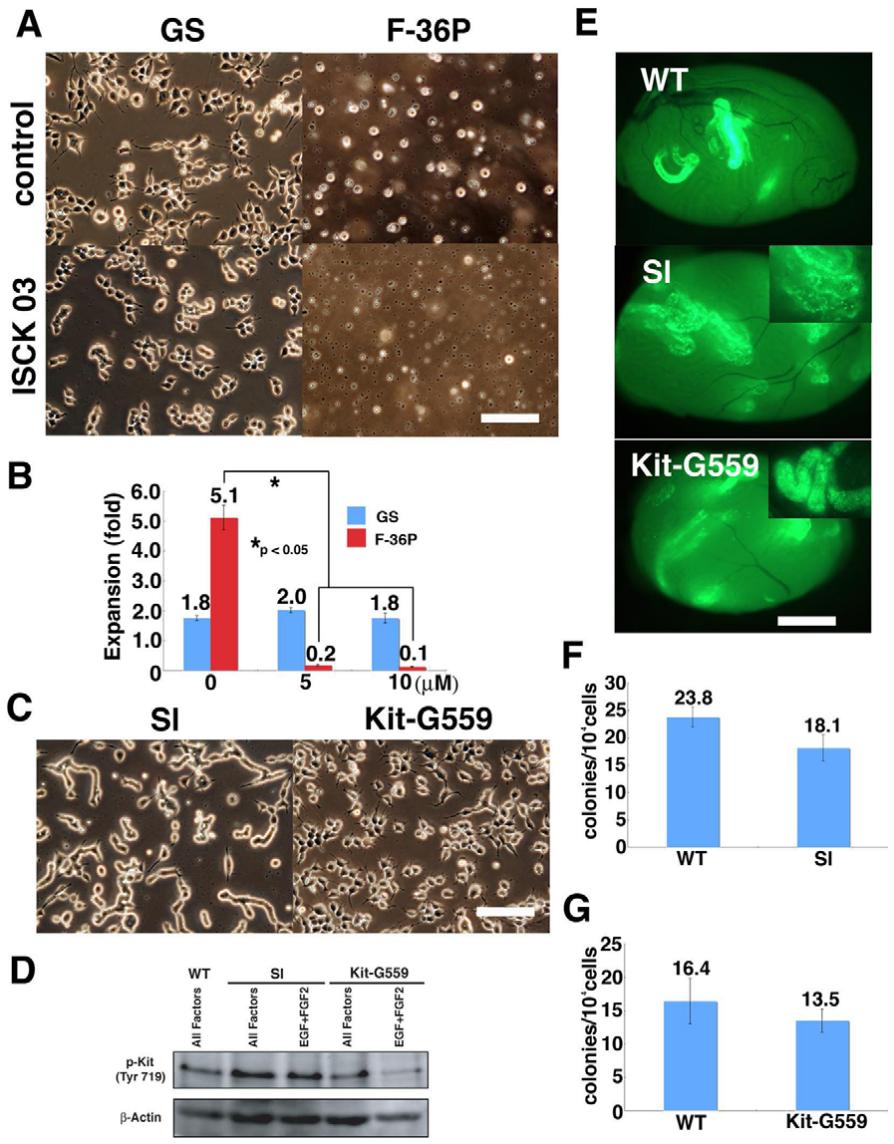
Dispensable role of Kit in GS cells. (A, B) Effect of Kit inhibitor (ISCK03) in colony morphology (A) and proliferation (B) of GS cells. Whereas the inhibitor could suppress the growth of the F-36P lymphocyte cell line effectively, no significant effect was found for GS cells. Cells were plated at 1×10^5^/3.8 cm^2^ and cultured with indicated cytokines for 5 (GS) or 3 (F36P) days. (C) Appearance of transfected GS cells. Note the elongated colonies of GS^Sl^. (D) Western blot analysis of transfected cells. GS^Sl^ showed an enhanced phosphorylation of Kit. (E) Macroscopic appearance of recipient testes that received transfected GS cells. Whereas GS^Kit-G559^ cells differentiated normally, GS^Sl^ cells proliferated on the basement membrane and no vertical differentiation was observed (inset). (F, G) Homing efficiency of transfected cells. Approximately 8×10^3^ cells were transplanted into each testis. No significant changes were induced by Kit-G559 (F) or Sl (G) transfection. Bar = 100 µm (A, C); 1 mm (E).

Although strong Kit expression in feeder-free culture conditions suggested that the undifferentiated state of SSCs is not maintained effectively, GS cells on laminin could be maintained for 6 months without losing SSC potential [15], which raised the possibility that Kit expression was correlated with SSC activity. To examine whether Kit is necessary for GS cell proliferation on laminin, we used a Kit inhibitor (ISCK03) to study the role of Kit in GS cells on laminin. Although the inhibitor prevented proliferation of control Kit-dependent F-36P leukemic cells in a dose-dependent manner [20], it did not show any effects on GS cells (Figure 2A and B). Addition of ACK2, a Kit neutralizing antibody, also did not influence GS cell proliferation (data not shown). These results agreed with the previous observation that Kit is dispensable for proliferation of undifferentiated spermatogonia [9]–[11]. On the other hand, we also examined whether Kit expression can promote GS cell proliferation. Different concentrations of soluble Sl (5 to 150 ng/ml) were added to the laminin culture, but the number of cells that recovered after a 5 day-period did not show a significant increase compared with control, and they maintained their fibroblastic morphology (data not shown).

Although these results suggested that Kit is dispensable in GS cell proliferation, it was still possible that soluble Sl did not provide a strong signal through Kit; it is known that membrane-bound Sl can activate Kit more strongly [21]. Indeed, Sl^d^ mutant mice, which lack the membrane-bound form of Sl, are deficient for spermatogenesis despite the expression of soluble Sl [12]. To overcome this problem, we stably transfected WT Sl and dominant active Kit cDNA (Val559 to Gly mutation; Kit-G559) into GS cells derived from an enhanced green fluorescent protein (EGFP)-expressing transgenic mouse [22]. While Kit-G559-transfected cells (GS^Kit-G559^) did not change morphology, Sl-transfected cells (GS^Sl^) produced elongated colonies and did not show flat appearances despite being plated at high cell density (Figure 2C). Although Western blot showed phosphorylation of Kit in WT and the transfected GS cells, the transgenes could not replace any of the cytokines used in GS cell culture (Figure 2D).

We further examined the effect of the transgenes in SSC colonization by germ cell transplantation [4]. GS^Sl^, GS^Kit-G559^ and GS^WT^ cells were transplanted into WBB6F1-W/W^v^ (W) mice, which lack endogenous differentiating germ cells [11]. Two months after transplantation, numbers of colonies in recipient testes were counted under UV light (Figure 2E). Although both transgenes did not influence SSC homing (Figure 2F and G), we noticed abnormalities in subsequent colony development. Interestingly, while GS^WT^ and GS^Kit-G559^ could differentiate normally, GS^Sl^ cells could not initiate vertical differentiation in the recipient testes (Figure 2E, inset), suggesting that regulation of Kit activation is critical for completing spermatogenesis. Thus, activation of Kit did not influence GS cell proliferation or SSC homing into the germline niche but has an impact on subsequent differentiation.

### SSC Activity of GS Cells with Kit Expression

**Figure 3:**
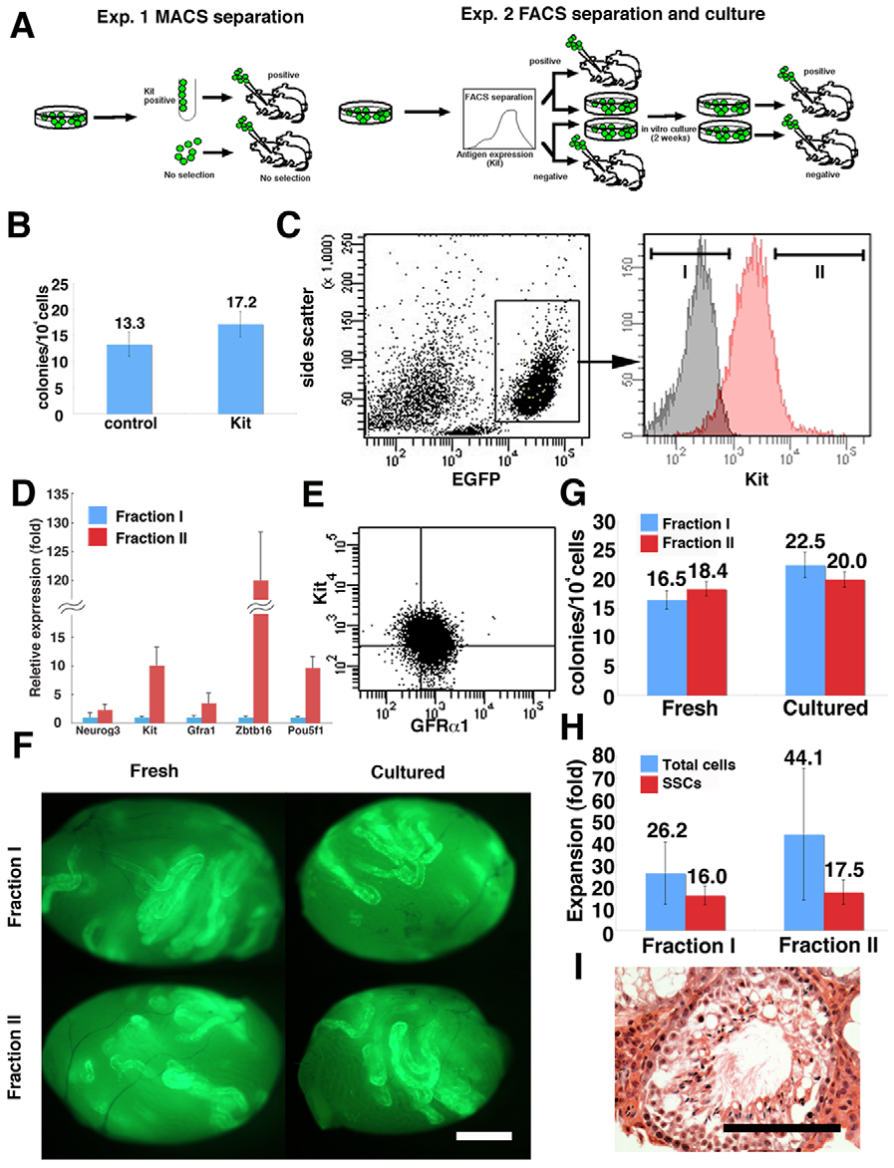
Fractionation of GS cells by Kit. (A) Experimental strategy. In the first experiment, Kit-expressing cells were selected by MACS. In the second experiment, GS cells were separated according to Kit expression levels by FACS. A portion of sorted cells was directly injected in each testis, and the rest of the cells were cultured for 2 weeks before transplantation. (B) SSC activity of MACS-separated cells. No significant difference was found. (C) Fractionation of GS cells by FACS. Distributions of stained (red) or control (black) are shown. (D) Real-time PCR analyses of sorted cells (n = 3–8). (E) Double immunostaining of GS cells by Kit and GFRa1. (F) Appearance of testes that received fresh and cultured cells. (G) SSC activity of fresh and cultured cells. No significant difference was found. (H) Increase in cell and SSC number after 2 weeks of culture. No significant difference was found. (I) Spermatogenesis in the recipient testis. Bar = 1 mm (F), 100 µm (I).

To directly test whether Kit-expressing GS cells on laminin can colonize seminiferous tubules, we used magnetic activated cell sorting (MACS) (Figure 3A). EGFP-expressing fibroblastic GS cells were selected by anti-Kit antibody, and were used for selection. After selection, 5.2±1.8% (n = 3) of the cultured cells could be recovered, and cells were then microinjected into seminiferous tubules of W mice. Two months after transplantation, analysis revealed that Kit-expressing cells have SSC activity. Whereas control unfractionated cells produced 17.2±2.4 colonies/10^4^ injected cells, Kit-expressing cells showed 13.3±2.3 colonies/10^4^ injected cells (n = 18). The value was not statistically significant (Figure 3B).

To extend this observation, we next used fluorescence activated cell sorting (FACS) to fractionate GS cells on laminin according to Kit expression levels (Figure 3A and C). We initially characterized sorted cells by real-time PCR for spermatogonia marker expression. Real-time PCR analysis confirmed a difference in Kit expression levels, and showed stronger expression of several SSC markers, including Pou5f1, Zbtb16, and GFRα1, in Kit^+^ cells (Figure 3D). Because GFRα1 is specifically expressed in A_s_, and A_pr_ undifferentiated spermatogonia in vivo and, therefore, the GFRα1^+^ population did not express Kit [3], we also checked expression patterns of GFRα1 at the protein level by flow cytometry. FACS analysis of GS cells showed that GFRα1 expression is found in both Kit^+^ and Kit^−^ cells (Figure 3E).

To compare proliferative potential, we cultured the sorted cells in vitro. Before initiating culture, cells from both fractions were microinjected into W mice directly to evaluate initial SSC content (Figure 3F). The remainder of the sorted cells was plated in culture for in vitro expansion. In these experiments, cells were plated on MEFs, because they promoted the survival of sorted cells more efficiently than did laminin possibly because of damage after sorting. In three sets of experiments, total cell numbers from both fractions expanded 8 to 55-fold during these 2 weeks of culture, regardless of Kit expression levels. After 2 weeks of culture, cells were transplanted into W mice to measure the increase in SSC numbers.

Analyses of recipient animals confirmed the results of MACS experiment; fresh Kit^+^ produced 18.4±1.2 colonies/10^4^ injected cells (n = 14), whereas Kit^−^ cells yielded 16.5±1.6 colonies/10^4^ injected cells (n = 17, Figure 3G). Differences between the two fractions were not statistically significant. Moreover, the concentration of SSCs in GS cell culture was also comparable after in vitro culture. Cultured Kit^+^ and Kit^−^ cells produced 20.0±1.3 and 22.5±2.2 colonies/10^4^ injected cells (n = 14), respectively. The overall increase in SSC number (SSC concentration × cell increase) in Kit^+^ and Kit^−^ cells was 17.5 and 16.0-fold, respectively, and the difference was not statistically significant (Figure 3H). Histological analysis confirmed normal spermatogenesis (Figure 3I). These results indicated that Kit^+^GS cells not only had SSC activity but also underwent self-renewal division at a level comparable to Kit^−^ GS cells.

### Changes in SSC Phenotype In Vivo

Finally, we examined whether SSCs undergo phenotypic changes in vivo. We hypothesized that active proliferation of SSCs might induce such changes and examined phenotypes of SSCs after germ cell transplantation. It is considered that SSCs expand in seminiferous tubules by increasing the probability of self-renewal division during the early phase of transplantation [23]. Three months after transplantation, however, transplanted cells establish a spermatogenic wave and produce spermatozoa.

**Figure 4:**
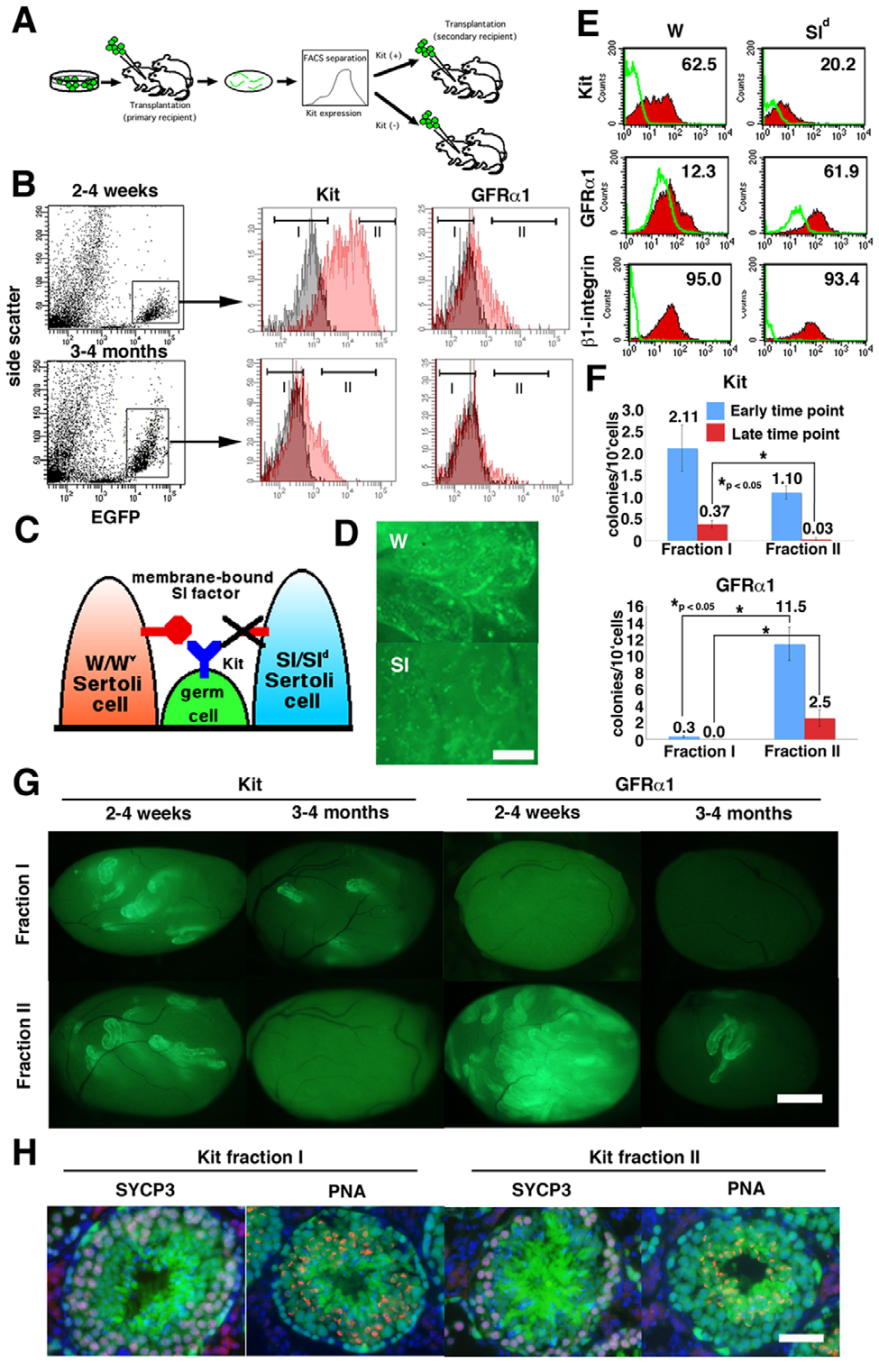
Changes in Kit expression in vivo. (A) Experimental strategy. After transplantation of GS cells, EGFP-expressing donor cells were fractionated according to Kit or GFRα1 levels. Sorted cells were transplanted into W mice. (B) Fractionation of donor spermatogenic cells. EGFP^+^ cells were gated and fractionated into two groups according to Kit or GFRα1 levels. Distributions of stained (red) or control (black) are shown. (C) Sl-Kit interaction in W and Sl^d^ mice. Germ cells in W mice have a defect in Kit and cannot respond to Sl, whereas Sertoli cells in Sl^d^ mice do not express membrane-bound Sl and cannot support differentiation. (D) Appearance of W and Sl^d^ recipient testes 2 weeks after transplantation. Differentiation was limited in Sl^d^ testis. (E) FACS analysis of W and Sl^d^ recipient testis after transplantation. EGFP^+^ cells were gated for analysis. (F) SSC activity of sorted cells. Both Kit^−^ and GFRα1^+^ cells showed significant enrichment of SSCs at both time points. (G) Appearance of recipient testes that received sorted cells. (H) Immunohistological section of the recipient testes that received Kit^+^ or Kit^−^ cells. The donor cells were collected from the primary recipient testes 2 weeks after transplantation, and the recipient testes were stained 2 months after cell sorting. The sections were stained with Rhodamine-PNA (red) for acrosomes and with anti-SYCP3 antibody (blue) for meiotic cells. Bar = 20 µm (D), 100 µm (G), 50 µm (H).

We microinjected EGFP-expressing GS cells into the seminiferous tubules of W mice (primary recipients). The recipient animals were sacrificed at early (2 to 4 weeks) and late (3 to 4 months) time points after transplantation, and single cells were obtained by enzymatic digestion (Figure 4A). Expression of Kit or GFRα1 in donor cells could be specifically analyzed by gating cells with an EGFP donor marker (Figure 4B), which was downregulated during meiosis [11]. Whereas EGFP^+^ cells showed a low side-scatter value in recipients at the early time point, they exhibited higher side-scatter value at late time point, indicating the progression of spermatogenesis [5]. Interestingly, development of this Kit^+^ population in recipients did not depend on membrane-bound Sl, because ∼20% of Kit^+^ cells were found when GS cells were transplanted into Sl^d^ testes (Figure 4C–E). On the other hand, Sl^d^ testes were enriched with GFRα1^+^ cells, suggesting that germ cells in Sl^d^ testes were relatively undifferentiated. No significant difference in β1-integrin expression was observed.

We fractionated the EGFP^+^ donor cells in the primary W recipient mice according to Kit or GFRα1 levels by cell sorting, and cells were retransplanted into seminiferous tubules of W mice (secondary recipients) to evaluate SSC activity. The number of colonies was smaller than were those from GS cells, suggesting that SSCs undergo more predominant differentiating divisions in vivo. Nevertheless, SSC activity was found in both Kit^−^ and Kit^−^ fractions when sorted cells were collected from recipients that had received donor cells within 4 weeks (Figure 4F and G). The number of colonies generated was 3.45±0.64 (n = 23) and 1.07±0.19 (n = 25)/10^4^ injected cells for Kit^−^ and Kit^+^ cells, respectively. Although the difference was statistically significant, SSCs expressing Kit were found in 5 of 6 experiments. In contrast, GFRα1^+^ cells were significantly enriched for SSCs, and results from three experiments showed that the numbers of colonies were 0.3±0.1 and 11.5±2.0/10^4^ injected cells (n = 15) for GFRα1^−^ and GFRα1^+^ cells, respectively (Figure 4F and G).

When sorted cells were collected from primary recipients between 3 and 4 months after transplantation, results from two experiments showed that the difference in SSC activity became more pronounced and the average numbers of colonies were 0.37±0.08 (n = 8) and 0.03±0.03 (n = 10)/10^4^ injected cells for Kit^−^ and Kit^+^ cells, respectively. In contrast, SSCs were consistently positive for GFRα1, and 2.5±1.0 colonies/10^4^ injected cells (n = 10) were generated only from GFRα1^+^ cells. Immunohistological staining of the recipient testes showed normal spermatogenesis from both Kit^−^ and Kit^+^ cells. No significant differences in SYCP3 (meiotic cell marker) or PNA (acrosome marker) expression patterns were observed (Figure 4H). These results show that SSCs also change Kit expression levels during regeneration in vivo.

## Discussion

Although both phenotypic and functional analyses suggested that most GS cells are progenitors without SSC activity, single-cell cloning experiments in our previous study showed that a significant proportion of GS cells maintain a potential to function as SSCs [16]. The current study was initiated to resolve the discrepancy between these findings, and we provide evidence that SSCs change their phenotype according to their microenvironment. Our conclusion was supported by our two transplantation experiments. First, in GS cell culture, Kit^+^ cells proliferated as actively as Kit^−^ cells and frequency of SSCs was comparable between the two populations. Second, immediately after transplantation, we found weaker but distinct SSC activity in the Kit^+^ donor cell population. These findings contrast with previous observations that SSCs do not express Kit. They also suggest that SSCs in vitro probably do not follow traditional scheme of SSC self-renewal [1], [2].

One of the important factors that contributed to phenotypic changes was laminin. Several lines of evidence have suggested that laminin plays critical roles in SSC biology. First, SSCs express both α6- and β1-integrin strongly and preferentially attach to laminin compared with other extracellular matrix substrates in vitro [12]. Second, β1-integrin-deficient SSCs that failed to attach to laminin could not settle in the germline niche [6]. Third, SSCs from mice, rats and hamsters all proliferate on laminin for several months without losing germline potential, suggesting that the ability to bind to laminin is beneficial and conserved among species [15], [24], [25]. Therefore, we speculated that integrin-laminin interactions in vitro might partly mimic stem cell-niche interactions in vivo, and assumed that culturing on laminin would create a more hospitable environment for SSCs. Given these results, we did not expect that GS cells on laminin would strongly upregulate Kit, a marker of differentiating spermatogonia.

Another factor that influenced SSC phenotype was plating density. Cell density or shape has been shown to influence many biological processes, including the lineage-specific marker expression or differentiation of stem cells. For example, changes in mechanical tension mediated by RhoA-ROCK signaling pathway regulated the fate commitment of mesenchymal stem cells (MSC)[19]. Dominant-negative RhoA committed MSCs to become adipocytes, whereas constitutive-active RhoA caused osteogenesis. Low plating density also enhanced their proliferation. In contrast, GS cells proliferated more slowly at low density, but cytochalasin D or transfection with dominant-negative RhoA reduced Kit expression, suggesting the involvement of actin cytoskeleton in Kit expression. This finding suggests the importance of cell structure and mechanics in the modulation of SSC phenotype and heterogeneity.

**Figure 5:**
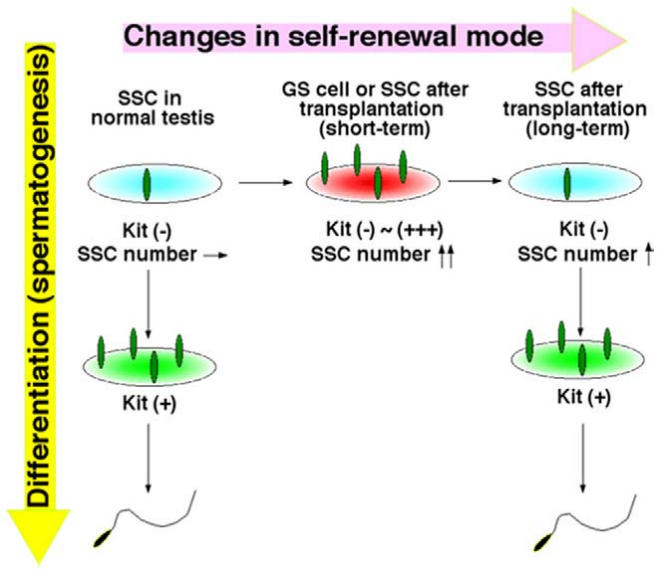
Expression of Kit on SSCs during active proliferation. SSCs in normal testes do not express Kit and maintain a constant number (non-activated state). However, when SSCs increase their number during culture or soon after transplantation, they upregulate Kit (activated state). Kit is downregulated in SSCs when germ cell colonies resume normal spermatogenesis.

Our retransplantation experiments showed that SSCs also change their phenotype in vivo. Retransplantation is a unique model to study SSC regeneration, because it allows SSCs to increase their number in vivo [26]. In normal testes, SSCs are kept under constant pressure to differentiate to produce sperm. SSCs undergo only two types of cell division, and they produce either two stem cells or two progenitor cells [1], [2]. However, the concentration of GDNF in the Sl^d^ or W testis is upregulated by a deficiency of endogenous germ cells [27], and this probably promoted transplanted SSCs to preferentially undergo symmetric self-renewal divisions to fill empty niches. Indeed, undifferentiated spermatogonia in Sl^d^ mice take up BrdU more rapidly than those in WT mice [27]. However, as SSCs gradually repopulate to establish normal cycles of spermatogenesis with time, the probability of self-renewing division progressively decreases by downregulation of GDNF and they no longer exhibit an activated phenotype. On the other hand, in other models used to study SSC regeneration, such as experimental cryptorchidism or vitamin A deficiency [5], [10], the number of SSCs remains constant, and this may explain why these treatments could not induce Kit in undifferentiated spermatogonia. Based on these observations, we suggest that, when SSCs are relieved from steady state kinetics, such as after germ cell transplantation or in vitro culture, they may be exempted from required differentiation and are induced to express Kit (Figure 5).

In some respects, our observation is reminiscent of the Clermont model of spermatogonial renewal, which proposes that A_1_–A_4_ spermatogonia, all of which express Kit, form a loop by recruiting a part of A_4_ into A_1_ [28], [29]. The loop proposed by this model is limited within A_1_–A_4_ spermatogonia, and SSCs are thought to divide only when there is a problem in A_4_ to A_1_ transition. This model opposes the single stem cell (A_s_) model, in which A_s_ spermatogonia differentiate unidirectionally. Although the experimental evidence favors the A_s_ model, studies in Drosophila initially showed that differentiated spermatogonia can dedifferentiate to form stem cells [30], and similar observations were also reported in mice. By taking advantage of lineage tracing, one study showed that undifferentiated spermatogonia that had already committed to differentiation reverted to SSCs [31]. Another study also showed that Kit^+^ differentiating spermatogonia in the “side population”, defined by the higher efflux of DNA-binding dye Hoeschst 33342, have SSC activity [32]. It will be interesting to study whether Kit^+^ cells also developed from progenitor cells in GS cell culture.

Since the development of germ cell transplantation technique, SSC phenotype was thought to be fixed, and SSCs have been isolated in deterministic manner. However, our analyses now show that phenotype of SSCs can change according to their microenvironment. Thus, caution is necessary when analyzing SSCs without functional assay. Because effects of enzymatic digestion on surface antigens cannot be excluded, different experimental approaches are required to test our hypothesis that activated and non-activated SSCs show distinct phenotypes. Identifying SSC-specific markers and factors that influence the mechanism of fate commitment in vitro will have important implications in studies of stem cells in other self-renewing tissues.

## Materials and Methods

### Ethics Statement

We followed the Fundamental Guidelines for Proper Conduct of Animal Experiment and Related Activities in Academic Research Institutions under the jurisdiction of the Ministry of Education, Culture, Sports, Science and Technology, and all of the protocols for animal handling and treatment were reviewed and approved by the Animal Care and Use Committee of Kyoto University.

### Cell Culture

GS cells used in the present study were derived from a transgenic mouse line C57BL/6 Tg14(act-EGFP)OsbY01 that was backcrossed to DBA/2 background. The method for GS cell culture using StemPro-34 SFM (Invitrogen, Carlsbad, CA) was described previously [13]. For laminin culture, GS cells were transferred on dishes that had been coated with 20 µg/ml laminin (BD Biosciences, Franklin Lakes, NJ) for 2 h at room temperature [15]. For transfection, cDNAs encoding mouse Kit-G559 (a gift from Dr. T. Tsujimura, Hyogo College of Medicine), and dominant-negative RhoA-N19 (a gift from Dr. D. M. Pirone, University of Pennsylvania) was cloned into pCAG-IRES2-neo, whereas cDNA mouse Sl (a gift from Dr. Y. Matsui, Tohoku University) was cloned into a CSII-EF-IRES2-puro lentivirus vector. Virus particles were produced by transient transfection of 293T packaging cells, as previously described [25]. Transfected cells were selected by 40–120 µg/ml G418 (Invitrogen) or 110 ng/ml puromycin (Sigma, St. Louis, MO)[16], [33]. ISCK03 was added at 1 or 5 µM (EMD Chemicals, San Diego, CA). F-36P cells (a gift from Dr. I. Matsumura, Osaka University) were maintained in RPMI supplemented with 10% fetal bovine serum (FBS). Increases in cell number were measured 5 days after initiation, whereas F-36P cells were cultured for 3 days.

### Animals and Transplantation

W and Sl^d^ mice were purchased from Japan SLC (Hamamatsu, Shizuoka, Japan). For transplantation of cultured cells, cells were incubated with 0.25% trypsin/1 mM EDTA to obtain single-cell suspensions. For serial transplantation, testis cells from primary recipients were dissociated at indicated time points with a two-step digestion method using type IV collagenase and trypsin (both from Sigma), as described [4]. Donor cells were introduced into seminiferous tubules of W or Sl^d^ mice via efferent duct (4–6 weeks old). Approximately 4 µl of the donor cell suspension could be injected. To avoid rejection of donor cells, recipient animals were treated with anti-CD4 antibody (GK1.5, gift from Dr. T. Honjo, Kyoto University), as described previously [34].

### Cell Staining and Selection

Dissociated cells were suspended (5×10^6^ cells/ml) in 1 ml of phosphate buffered saline containing 1% FBS (PBS/FBS). Cells were then incubated with primary antibodies for 20 min on ice, washed twice with PBS/FCS, and used for cell separation. Primary antibodies used in this study were anti-rat GFRα1 (81401; R&D systems, Minneapolis, MN), R-phycoerythrin (PE) or allophycocyanin (APC)-conjugated rat anti-mouse Kit (2B8; BD Biosciences), APC-conjugated anti-rat α6-integrin (GoH3; BioLegend, San Diego, CA), anti-mouse E-cadherin (ECCD2; Takara Biomedicals, Shiga, Japan) and biotinylated anti-mouse β1-integrin (Ha2/5, BD Biosciences). For MACS, cells were further incubated for 20 min with Dynabeads M-450 sheep anti-rat IgG (Invitrogen) with agitation, and target cells were separated according to the manufacturer's protocol. For flow cytometric analysis and sorting, APC-conjugated streptavidin, and ant-mouse or -rat IgG (all from BD Biosciences) were used as secondary reagents. After the final wash, 1 µg/ml of propidium iodide was added to samples to eliminate dead cells. Stained cells were analyzed by FACSCalibur or sorted by FACSAria II (both from BD Biosciences).

### Analyses of Recipient Testes

The number of colonies was counted under a stereomicroscope equipped with UV light. We defined a donor cell cluster as a colony when it occupied the entire basal surface of the tubule and was longer than 0.1 mm. For immunohistological staining, the recipient testes were fixed in 4% paraformaldehyde and then frozen in Tissue-Tek OCT compound (Sakura Finetechnical, Tokyo, Japan) for cryosectioning. The slides were analyzed under confocal laser scanning microscopy. Meiosis was detected by immunofluorescence using anti-synaptonemal complex protein 3 (SYCP3) antibodies, which was prepared in our laboratory using a synthetic oligopepetide [35]. The anti-SYCP3 antibody was detected by Alexa 488-conjugated anti-rabbit immunoglobulin G antibodies (Molecular Probes, Eugene). Rhodamine-conjugated Peanut agglutinin (PNA) was used to detect acrosomes (Vector, Burlingame, CA). For preparation of paraffin slides, testis samples were fixed in 10% neutral-buffered formalin and processed for paraffin sectioning. Sections were stained with hematoxylin and eosin.

### Western Blot Analysis

Samples were separated by SDS/PAGE, transferred to Hybond-P membranes (Amersham Biosciences, Buckinghamshire, UK), and incubated with anti-phospho-Akt (Ser 473) or anti-phospho-c-kit (Tyr 719) antibody. After washing, peroxidase-conjugated anti-rabbit IgG was used as the secondary antibody (all from Cell Signaling, Danvers, MA).

### Real-Time PCR

Total RNA was isolated using Trizol reagent (Invitrogen). First-strand cDNA was synthesized using Superscript^TM^ II (RNase H^−^ reverse transcriptase, Invitrogen). For quantification, StepOnePlus^TM^ Real-Time PCR system and *Power* SYBR Green PCR Master Mix were used according to the manufacturer's protocol (Applied Biosystems, Warrington, UK). Transcript levels were normalized to those of Hprt1. PCR conditions were 95°C for 10 min, followed by 40 cycles at 95°C for 15 s, and 60°C for 1 min. Experiments were performed on each subpopulation purified from three independent sorting experiments. Each PCR was run at least in triplicate using specific primers (Table S1).

### Statistical Analysis

Results are presented as mean±SEM. Data were analyzed by Student's *t*-tests. Significant difference in the ISCK03 effect was determined by Tukey's HSD multiple comparisons test.

## Supporting Information

Table S1 Real-time PCR primers used in the experiments.
(0.04 MB DOC)
