# Supplementary material for: Phenotypic Plasticity of Mouse Spermatogonial Stem Cells
Source: PLoS One. 2009 Nov 19;4(11):e7909. doi: 10.1371/journal.pone.0007909 (PMC2774941; doi:10.1371/journal.pone.0007909)
Supplement: Table S1 — Real-time PCR primers used in the experiments. (0.04 MB DOC) [file pone.0007909.s001.doc]

| **Genes** | **Primer sequences** |  |
| --- | --- | --- |
|  | **Forward** | **Reverse** |
| HPRT | GCTGGTGAAAAGGACCTCT | CACAGGACTAGAACACCTGC |
| Neurog3 | AGCGGACCACAGCTTCTATG | AGATGCTTGAGAGCCTCCAC |
| Kit | ACTCCAACTTGGCAAACTGC | CGCTACCCTGGAATAGGATG |
| Gfra1 | GGACCGCCTGGACTGTGT | GCTGCAGCTCTGTTCCTTCAG |
| Pou5f1 | CCCAACGAGAAGAGTATGAGG | TGATCAACAGCATCACTGAGC |
| Zbtb16 | ACCAGTGTACCATCTGCACG | TGATAGCCAGATCACCTGGAG |
